# To Young Practitioners

**Published:** 1883-05

**Authors:** 


### TO YOUNG PRACTITIONERS.

Dentists of age, experience and reputation, are oftentimes in the receipt of letters from young practitioners, inquiring what instruments, materials, or remedies they employ, in the hope that by using the same they will reach like results. A young dentist sees a beautiful filling in the mouth of a patient, and enquires who was the operator. On being informed that it is Dr. A.'s work, he writes the experienced practitioner asking what gold he used, what instruments, and if the filling was inserted by mallet or hand pressure. He receives a courteous answer, and is told that crystal gold was employed, and the filling consolidated by hand pressure. He tries the same methods and materials, but his work proves an utter failure. Applying in this extremity to Dr. B., who is celebrated for his operations, he is assured that crystal gold is a humbug, and is favored with a long lecture upon the uses of the mallet. Straightway the seeker after ready-made excellency casts aside all his old methods and instruments, procures a set of the latest agony in mallet pluggers, some special foil, and goes to work, only to find that in his hands the new method is as complete a failure as the other, and down goes his head in the bulrushes again. But hope springs eternal in his student breast, and hearing much of the wonderful work of Dr. C., away he goes on another pilgrimage, in search of a short cut to glory. Dr. C. tells him that the mallet is a mocker, and soft gold is raging, and whosoever is deceived thereby is not wise. Electricity, cohesive gold, and contour ; these are the things my boy, and through them you may reach your goal. He invests in a battery, becomes familiar with bichromate of potash and carbon plates, only to discover that true excellence does not lie in sulphuric acid or rolled zinc.

And so the zealous and foolish student in search of a royal road to eminence, will seek to take ready-made the experience of others, make some patent process supply his lack of patience, a gimcrack ingenuity answer instead of preseverance, and finally to rise to the summit of his profession by sheer empircism and charlatanry. If he has sufficient ability it is ten chances to one that he takes a New Departure, flings to the winds all the long treasured wisdom experience and dexterity of generations, and adds some new patent to the long list of tomfoolery that has been inflicted upon a suffering profession. Beginning with an honest and earnest desire to become skillful, he takes the wrong road, and finally, discouraged and disheartened by his inability to find the goal of his ambition, he turns quack and sets up a toll-gate at the entrance of a new path to professional perdition.

Long ago Euclid declared that there is no royal road to Geometry. The young practitioner who proposes to reach the front rank has cut out for himself a rough path, but if he has some ability and a great deal of industry and perseverance his success is certain, because the majority of young men are lazy, and propose to try a short cut, while it is only those who toil painfully around the rough road, creeping every foot of the way, that reach the summit. The expert manipulator, who has by years of the hardest kind of labor made every step familiar, is not particular about what gold he uses. His knowledge of methods and his manual dexterity are such that he can produce perfect results with almost any good instrument, and in the absence of these he will fashion a plugger out of a rusty nail, and with this will secure triumphs unknown to the inexpert. A good fisherman will catch fish with a bent pin, while the awkward novice will flourish his bamboo rod and cast his patent fly in vain.

One of the most eminent surgeons of to-day performed the Cæsarean operation upon a backwoods patient, with only a jack-knife for a scalpel. Many miles from his office, without an instrument case, he operated with what implements the house afforded, and saved both mother and child. But his success should not inspire another to ignore all proper tools and fit appliances, and lead him to depend upon improvised substitutes. The happy tissue of the case was due to the surgeon's knowledge and experience, and these were the fruits of long years of hard labor and exhaustive study. They took the place of the proper utensils, and they were sufficient; but the surgeon who has neither experience nor instruments, is scarce clear of the crime of murder if he unnecessarily attempts an operation.

The young dental practitioner, fresh from college, just entering upon practice, encounters obstacles which he is sometimes little prepared for. He has learned to consolidate gold in an extracted tooth held in a vice. Under the watchful care of an experienced demonstrator he has worked upon the living patient, and has been made familiar with the theory of operative dentistry. But when removed from the superintendence of a master he attempts an independent practice, and his operations are submitted to the test of time, he has the mortification of seeing his proudest achievements fail, and the fatal blue line at the margin of fillings teaches him that there is a mistake somewhere. He has worked precisely as he was taught, and his freshly-completed operation looked quite as well to his comparatively inexperienced eye, as that of his teacher. He does not see the microscopic imperfections, and in his perplexity he perhaps seeks refuge from an acknowledgement of his manipulative inability in some such subterfuge as "incompatibility," or "chemical erosion." If he will go back over the ground again and again, until experience has made him a competent judge, if he will be entirely honest with himself, he will no longer endeavor to place the responsibility for his failures where it does not belong.

The material used is a secondary consideration in the saving of teeth. Skill equal to the occasion, and uncompromising faithfulness are the first requisites, but these premised, of course he can secure better results with a good material than with one that is inferior. If any one would learn the secret of the success that attended upon such operators as Varney and Webb, it is here, in a nutshell: skill, the result of a long and painful experience, and faithful, honest, laborious work. Both of these eminent operators had wrought a hundred times harder in acquiring their manipulative ability than they did in performing an operation afterwards. Both fell victims to their unsparing faithfulness and their untiring fidelity, but both left behind them reputations that should be an inspiration to every honest dentist.

Neither was without failures, but when such were detected neither asserted his own infallibility, and attempted to throw the blame upon the materials used, or the environments of the case. Both may have had faults, but they were free from that most monstrous one of attempting to deny them.

> "The fault, dear Brutus, is not in our stars,
> But in ourselves, that we are underlings."

There is hope for the dentist who acknowledges his errors and endeavors to profit by his mistakes, but for him who would screen himself from deserved censure by attributing his failures to anything but his own slovenliness, the really honest man can entertain no other feeling than that of contempt. Every experienced dentist has seen the most enduring fillings made of comparatively poor materials. Go into the office of one of the older dentists, who acquired his skill and made his reputation before the advent of the modern machinery that is so indispensable to the younger operator, and you will doubtless find him turning out the most beautiful and perfect operations with appliances which seem totally inadequate. Long experience has made him the master of the tools he uses, and he wisely declines to attempt to learn new tricks. For his trained hand and laborious faithfulness modern methods and improvements are unnecessary. But all the cunning implements fashioned by the hand of man could not take the place of that experimental knowledge, that serious, stern determination to do his best, that has made him what he is. If there be upon earth one spectacle more sublime than another, it is that of the sturdy, honest, faithful dentist, who gives to every operation the very best fruits of his ability, and through a long life renders to every patient the full return for that which he receives. There are so many temptations to dishonesty in our profession that it is little wonder that few succeed. It is so much easier to putty up a tooth with some patent compound than it is to insert a thorough gold filling, perfect at every point, that young men who love their ease seldom turn out good operators in foil. Rubber dentures are so readily inserted, and afford such opportunities for the indulgence of indolence, that the race of gold-plate workers has become almost extinct. If one would find an honest executor of his estate, let him seek for an expert in gold work, for it necessarily follows that the man who is honest with himself, and faithfully laborious in his profession, would not prove recreant when other interests were entrusted to his keeping.

We are not pleading in this article for the use of gold. There is room for conscientiousness in the employment of other materials, but while slovenly work may for a time pass muster with them, gold more quickly betrays the careless dentist. Our original proposition was that teeth are not saved by any particular method or material, but by intelligently directed, hard work. A great painter was once asked with what he mixed his pigments. "With brains," was the answer, and the successful dentist might return the same when asked with what he filled teeth. If a visit be made to the lumber room of almost any dentist who has been years in practice, there will be found a collection of curious contrivances and discarded devices, accumulated in his earlier years, in the vain attempt to make intricate machinery or mechanical ingenuity take the place of laborious skill. He has long since learned the futility of all such efforts, and as a consequence they are banished from his work rooms. But dentists of the next generation are still busily engaged in the same hopeless task, until they too are taught by a like bitter experience. Without the knowledge to properly use them, they procure power engines and electric pluggers, and when the work done with them fails, the blame is thrown upon the implements, and they are cast aside as worthless. It is criminal for the experienced dentist to advise the tyro to commence his career by any attempt to rely upon machinery, until he shall first have fully learned to do his work without them. When this is accomplished, he may safely procure them as aids to lighten his labors, and he will be enabled to intelligently employ them without injury to the tissues which they might otherwise destroy.

Teeth, then, may be saved with amalgam, with gold, with gutta-percha, with tin, but not *by* either of these materials. It is intelligently directed skill, and faithful, honest, persevering hard work, that makes the really successful operator, and the young practitioner cannot too early or too thoroughly have this fact indelibly impressed upon his mind.

